# Living by the sea: place attachment, coastal risk perception, and eco-anxiety when coping with climate change

**DOI:** 10.3389/fpsyg.2023.1155635

**Published:** 2023-06-22

**Authors:** Natacha Parreira, Carla Mouro

**Affiliations:** ^1^Iscte-Instituto Universitário de Lisboa, Lisbon, Portugal; ^2^Centro de Investigação e Intervenção Social, Iscte-Instituto Universitário de Lisboa, Lisbon, Portugal

**Keywords:** place attachment, risk perception, eco-anxiety, coping strategies, trust in authorities, coastal areas, Aveiro

## Abstract

Climate change poses major threats to coastal regions. In Portugal, the Aveiro district is one of the most vulnerable areas due to urbanized areas’ exposure to the dangers of rising water. The prospect of flood threats can trigger a range of cognitions and emotions that affect adaptation and mitigation measures’ success. This study sought to examine whether active and traditional place attachment is associated with residents’ active and passive coping strategies to deal with the risk of rising water levels. An additional aim was to clarify whether these relationships are mediated by risk perception and eco-anxiety. The links between individuals’ level of trust in authorities and coping mechanisms were also examined. An online questionnaire was completed by 197 Aveiro residents. The data show that active place attachment is connected to greater risk perception, eco-anxiety, and adoption of active coping strategies (e.g., problem solving). Low eco-anxiety was also found to have a positive effect on active coping strategies. Lower trust in the responsible authorities was additionally associated with active coping mechanisms. Overall, the results support the sequential mediation model for active coping but not for passive coping. The findings reinforce the need to consider cognitive factors (e.g., risk perception) and emotional factors (e.g., place attachment and practical eco-anxiety) to understand more fully how coastal residents deal with flood threats. Practical implications for policymakers are discussed.

## 1. Introduction

In 2018, the United Nations published the Intergovernmental Report on Climate Change based on research into the impact of a 1.5° centigrade rise in the planet’s temperature, warning of the urgent need to reduce carbon dioxide (CO_2_) emissions. Climbing temperatures are driven by CO^2^ levels in the atmosphere, and thawing glaciers contribute to rising water levels, which pose major threats to coastal regions and communities. Despite these risks, people feel attracted to coastal areas, so these zones are characterized by high productivity and population growth (Lloret et al., 2008). By 2030, 50% of the population is expected to reside within 100 kilometers (km) of the coast (Small and Nicholls, 2003), thereby increasing these regions’ socioeconomic vulnerability. Aveiro has been flagged as one of the most at-risk coastal areas in Portugal (Kulp and Strauss, 2019). Rising water levels in this low-altitude region in northern Portugal could have a severe impact on existing habitats and local communities’ livelihoods (Luís et al., 2016).

Despite growing concerns worldwide about mounting exposure to environmental threats, the “slow scale” (Sullivan and Young, 2020) of disasters’ occurrence helps explain the marked variability of how people perceive and deal with this problem. On the one hand, debates, protests, and mentions in the news have increased, suggesting individuals’ greater involvement in and increased stress and anxiety about the issue. On the other hand, people living near the coast are aware of climate change’s effect, yet they still appear to minimize their region’s risk and to be less concerned and prepared (Costas et al., 2015; Domingues et al., 2021). Coastal residents thus often resist the responsible authorities’ policies and mitigation plans (Goeldner-Gianella, 2007; Goeldner-Gianella et al., 2015).

To deal with future threats, individuals use various coping strategies (Folkman and Lazarus, 1991), namely, psychological adaptative mechanisms focused on reducing external or internal conflicts. These responses are the outcome of each person’s assessment of their relationship with the environment, and the mechanisms can be both behavioral and cognitive (Lazarus and Folkman, 1991). People’s coping strategies can be more active or passive and inhibit or encourage behavioral change (Folkman and Lazarus, 1988; Lazarus and Folkman, 1991; Homburg et al., 2007; Lemée et al., 2019). Individuals’ diverse responses can play a decisive role in their communities’ adaptation to climate change. If residents feel informed and empowered to help control threats, they can work with the relevant agencies. If locals feel unable to deal with the problem, they may, for example, respond with denial (i.e., treating the issue as a distant problem), avoid negative emotions, or stay away from the topic (Ojala and Bengtsson, 2019; Sullivan and Young, 2020).

It is thus crucial to better understand the psychological mechanisms underlying residents’ choice and implementation of coping strategies. Threat assessments are influenced by cognitive responses, such as risk perception (Stancu et al., 2020) and trust in authorities (Siegrist et al., 2005), and affective reactions, such as fear, anxiety (Siegrist et al., 2005; Homburg et al., 2007; Terpstra, 2011), and place attachment (de Dominicis et al., 2015; Sullivan and Young, 2020).

Place attachment reflects the intensity and psychological nature of individuals’ relationship with their place of residence, manifested as affective, cognitive, and behavioral processes (Devine-Wright, 2011; Sullivan and Young, 2020). Place attachment has traditionally been studied as a factor that influences how people perceive risks and which coping strategies they adopt (de Dominicis et al., 2015; Stancu et al., 2020; Casakin et al., 2021; Domingues et al., 2021). Researchers have detected both positive and negative impacts of place attachment (de Dominicis et al., 2015; Bonaiuto et al., 2016; Casakin et al., 2021), which underlines the need to understand more deeply these effects’ role in efforts to manage climate change threats.

In addition, many scholars have conducted a one-dimensional analysis of place attachment, that is, comparing stronger and weaker attachment (Hidalgo and Hernández, 2001; Casakin et al., 2021), often not considering that people can establish strong attachment to places across distinct dimensions (Lewicka, 2011; Devine-Wright and Quinn, 2021). The inconsistent findings on this variable’s role may, therefore, be due in part to a lack of acknowledgement of place attachment’s complexity and multidimensionality (Lewicka, 2011; Sullivan and Young, 2020; Casakin et al., 2021; Devine-Wright and Quinn, 2021). The present study sought to fill this gap by investigating how two types of place attachment—active and traditional—are related to coastal residents’ varied coping strategies.

This research looks at risk perception as a mediator that links place attachment and adaptive coping mechanisms. People with strong place attachment seem to perceive more risk (Bernardo, 2013), but studies in coastal areas have shown that individuals with stronger traditional place attachment show no desire to participate in risk reduction measures, a response associated with their distrust of the authorities and relativization of risks (Martins et al., 2009; Domingues et al., 2021).

Persistent concerns about future threats and uncertainties associated with climate change can also generate distress about future uncertainties, which has been defined as eco-anxiety (Kurth and Pihkala, 2022) or climate anxiety (Clayton, 2020). This anxiety is associated with the anguish caused by on-going concerns about climate threats and impending risks (Pihkala, 2020). Recent research has, however, highlighted that eco-anxiety leads to practical solutions that build more sustainable, resilient lifestyles (Grose, 2020; Pihkala, 2020; Kurth and Pihkala, 2022).

The present study focused on the sequential relationships between active and traditional place attachment, risk perception, eco-anxiety, and coping strategies. More specifically, this research sought to contribute to the existing literature by addressing the following questions:

- What roles do different types of place attachment play in the adoption of active and passive coping strategies?
- How are these connections affected by perceived risk and eco-anxiety?

This investigation further examined trust in authorities’ effect on individuals’ adoption of coping strategies.

## 2. Theoretical framework and hypothesis development

### 2.1. Aveiro and rising water levels

In Portugal, the Aveiro district is one of the main areas that could be affected by rising water levels (Kulp and Strauss, 2019). Between 1976 and 2003, the average sea level rose by 1.15 ± 0.68 millimeters per year, which strongly affected coastal erosion (Lopes et al., 2013). Aveiro is characterized by high population density in quite low coastal areas and intensely urbanized areas with agricultural fields near lagoon zones, such as the Aveiro estuary and the Vouga River. These patterns make this region and its population extremely vulnerable to climate change’s effects.

The district’s most prominent characteristic is the Vouga River’s convergence with the Ria de Aveiro in the Baixo Vouga Lagunar area, with shallow saltwater zones influenced by both tides and fresh water (ADAPT-MED, 2015). The Ria de Aveiro is approximately 45 km long and 10 km wide, and it crosses the Aveiro city center and connects the Baixo Vouga Lagunar area with the Atlantic Ocean (Luís et al., 2016). This region is quite prone to river floods due to heavy rains, among other factors (Lopes et al., 2013). Higher water levels can also be caused by extreme maritime events (e.g., live tides and storm surges) combined with intensive rainfall, which can have varied impacts such as floods, coastal erosion, contaminated drinking water, agricultural land salinization, and loss of habitats (Luís et al., 2016).

Varied initiatives have been created to minimize these events’ impact, notably the north jetty’s extension on Barra beach in Ílhavo and an Action Plan for Adaptation to Climate Change in 2021. The Aveiro Municipality has made the latter plan available to the public as it brings together varied adaptation measures that address climate change. These efforts include increasing residents’ awareness of and knowledge about their exposure to extreme weather events and these incidents’ effect on goods and people’s safety. Measures will also be taken to adapt infrastructure to cope with events such as floods, including the restoration of streams, recovery of water retention structures, improvement of streamflow conditions, and promotion of water reuse systems. These and other adaptation and mitigation procedures’ success is largely dependent on communities’ support and adherence, which means the authorities need to better understand how individuals cope with potential local danger.

### 2.2. Coping strategies

People develop coping strategies to deal with threatening events that can include cognitive, emotional, and behavioral processes, such as psychological adaptative mechanisms that control, tolerate, or reduce conflicts between external and internal demands (Folkman and Lazarus, 1991). Individuals thus adopt the strategies they have available or consider the most appropriate ones. Coping mechanisms are selected based on assessments of threats’ probability and their potential harm to valued property or people. If threats are perceived as real, individuals then evaluate their ability to cope with—or avoid being harmed by—these stressful situations (Lazarus and Folkman, 1991; Lemée et al., 2019; Ojala and Bengtsson, 2019) by using both constructive and defensive strategies (Sullivan and Young, 2020).

The same climate change threat can be perceived by one person as an obstacle and by another as a challenge to overcome (Mah et al., 2020), so a distinction must be made between the kinds of strategies adopted. The extant literature contains classifications of different types of coping mechanisms that distinguish between strategies focused on solving the problem or minimizing the resulting emotions (Lazarus and Folkman, 1991). A consensus has been reached on defining two major types of mechanisms: active coping and passive coping strategies (Nielsen and Knardahl, 2014; Lemée et al., 2019).

Active coping refers to ways to maintain surveillance of the situation by defining problems and actions to minimize or solve those issues (Lemée et al., 2019; Navarro et al., 2021). These mechanisms can include problem-focused and emotion-focused strategies, such as self-protection and problem solving that comprises seeking information about and planning or directing participation in climate-change adaptation measures (Homburg et al., 2007; Stancu et al., 2020).

Passive coping, in contrast, is characterized by strategies focused on non-involvement usually through passive or minimally adaptive responses (Nielsen and Knardahl, 2014) and on the reduction of negative feelings and emotions (Lemée et al., 2019) through changes in perceptions of threats. Some mechanisms are relativization (e.g., treating the risk as a future problem), denial of guilt (e.g., considering the problem as separate from personal actions), and positive thinking (e.g., trusting that someone else will solve the problem, trying to stay calm, and maintaining normal routines) (Homburg et al., 2007; Nielsen and Knardahl, 2014; Sullivan and Young, 2020).

People’s affective relationship with their place of residence (i.e., place attachment) can affect how individuals and communities choose between different coping strategies. This effect is discussed in greater detail in the next subsection.

### 2.3. Place attachment and coping strategies

Place attachment can be defined as people’s affective link to a specific place, neighborhood, community, or city, including a desire to remain close to that area because it conveys security and trust (Hidalgo and Hernández, 2001). Personal experiences and symbolic, emotional, and social connections cause individuals to acquire a sense of belonging and purpose associated with a place, linking it with their personal identity and well-being (Lemée et al., 2019; Sullivan and Young, 2020). Stronger place attachment has been associated with greater civic activity, mediated by local, social, and cultural factors (Lewicka, 2011).

Prior studies have highlighted the importance of place attachment to the way people deal with climate change’s impacts, namely, coping with weather-related disasters (Ruiz and Hernández, 2014), adopting preventive behaviors (de Dominicis et al., 2015), or accepting adaptation projects (Devine-Wright, 2011). Sullivan and Young (2020) argues that “if people are more emotionally connected to their environment, they should be more informed and vigilant about potential threats” (p. 6) and thus seek to deal more actively and adaptively with climate change. Other scholars found that when people deal with climate change, a strong place attachment seems to be positively correlated with more optimistic outlooks of the future, even for those experiencing solastalgia (Phillips and Murphy, 2021). This in turn leads to higher acceptance of adaptation measures and resilience.

However, other researchers have reported evidence suggesting that stronger place attachment can also negatively influence intentions to deal directly with climate change threats. Research on floods in Faro and Aveiro revealed that residents with higher levels of “traditional” place attachment were willing to make preparations to face potential disasters but refused to relocate to safer places despite the imminent threat (Martins et al., 2009; Domingues et al., 2021).

Individuals create relationships with and symbolism of the place where they live, which can increase their resistance to accepting environmental management measures (Devine-Wright, 2011). Various authors have proposed a typology of the affective relationships that people establish with their place of residence, which in turn are associated with distinct reactions to threats (Sullivan and Young, 2020) and the coping strategies adopted. Previous studies have confirmed that long-term residents’ place attachment is inherited through an intergenerational transmission of memories and that this kind of connection is paired with resistance to accepting disaster prevention measures (Domingues et al., 2021). Other research has revealed place attachment can arise from an active sense of community that leads to involvement in local measures focused on climate changes’ consequences (Paton et al., 2001). However, the exact nature of this relationship is unclear possibly because of place attachment’s complexity (Sullivan and Young, 2020).

Thus, a better understanding is needed of this attachment’s different dimensions and its relationship with the perceptions and emotions that contribute to individuals’ adoption of diverse coping strategies. Based on Hummon (1991) and Lewicka (2011), two place attachment dimensions were identified. The first is traditional attachment, defined as an inherited sense of place (Lewicka, 2011, 2013) associated with long-term residence in a family home. This type of bond is also linked with more religious and conservative values, a strong connection to the surrounding neighborhood, identification with the place through family tradition, and a greatest resistance to leaving that location (Lewicka, 2011).

The second dimension is place discovered attachment, connected with a long period of residence in a chosen place and with higher social and cultural capital and more active involvement with the community and institutions through civic participation or social capital contributions (Lewicka, 2011, 2013; Sullivan and Young, 2020). Given the latter dimension’s characteristics, the present study designated this type of link as active attachment.

Lewicka (2011) further identified a third dimension related to a highly mobile lifestyle and weak affective relationship with the place of residence. The latter was not included in the current research because this dimension is associated with quite low involvement with the place and in the surrounding community (Lewicka, 2011; Sullivan and Young, 2020).

Various studies reported in the environmental literature have concentrated on place attachment and coping strategies’ relationship, but few scholars have examined the association between different types of place attachment and diverse coping strategies. One exception is an investigation conducted in Italy and Romania that found evidence of a connection between diverse place attachment dimensions and coping strategy styles (Stancu et al., 2020). The attachment dimensions studied were based on the literature on interpersonal attachment (i.e., safe, anxious, and avoidant attachment). Stancu et al.’s (2020) results show that residents with a more active attachment style are more likely to use active coping mechanisms, and residents with avoidant attachment tend to have fewer social ties in the community and to adopt more passive coping strategies.

Based on the above findings, the present research included the following hypothesis:

*H1*: Different types of place attachment (i.e., active vs. traditional) are associated with different types of coping strategies.

*H1a*: Active place attachment is associated with more active coping strategies.

*H1b*: Traditional place attachment is associated with more passive coping strategies.

### 2.4. Place attachment, risk perception, and coping strategies

Risk perception is defined as an intuitive, situational assessment of risks made by individuals faced with uncertainties and limited information when interpreting specific threats (Slovic, 1987). Perceived risk is not constant over time as it reflects the relationships between threat awareness, concern, and preparedness. When one of these components increases, the perceived risk grows, but resilience increases as a result (Raaijmakers et al., 2008).

The literature on risk perception in coastal communities exposed to flood risks provides evidence that factors such as risk exposure frequency, past experiences, and place attachment affect risk perceptions (Slovic, 1987; Lemée et al., 2019). For instance, locals may ignore events that are less likely to occur even if their impact could be catastrophic (de Dominicis et al., 2015). Residents of places that have never had natural disasters are more likely to report a lower chance of these events occurring in their area (Domingues et al., 2021). People also appear to minimize flood risks shortly after an inundation has occurred, so these individuals are often resistant to adopting preventive behaviors to deal with future floods (de Dominicis et al., 2015).

Place attachment can function as a driver of or barrier to various cognitive, emotional, and behavioral processes related to the specific locality (Knez, 2005; de Dominicis et al., 2015). Individuals with strong place attachment tend to be more aware of environmental risks that threaten their zone (Bonaiuto et al., 2016), but other authors have reported that a strong attachment may actually reduce awareness of existing problems or dangers (Domingues et al., 2021). For example, individuals with a strong bond can avoid risk assessment to avoid the distress of dealing with threats, which contributes to denial and reduced risk perception (de Dominicis et al., 2015). Another potential explanation for this pattern may be that people establish different place attachment styles (i.e., active and passive) that can trigger different levels of risk perception. The latter reason is in line with Lazarus and Folkman’s (1991) transactional model of stress and coping, which states that, in stressful situations linked to perceived risks due to a known threat, individuals opt for specific coping strategies.

Risk perception has further been studied as a predictor of coping strategies. An investigation in China (Xu et al., 2018) found that people with higher risk perception report adopting more disaster reduction measures. This behavior has a direct effect on these threats’ consequences (Xu et al., 2018). Risk perception can thus influence residents’ willingness to participate in risk reduction strategies and improve their preparedness for potential disasters. Various scholars, however, have called for more multifaceted studies to explain the relationship between perceived risks of coastal flooding and locals’ willingness to deal with these threats (Lemée et al., 2019).

Risk perception is defined by both individual and situational characteristics and is associated with person-place variables, so the present research explored risk perception’s mediation of the relationship between place attachment and coping strategies. Higher levels of risk perception can have a positive effect on individuals’ intention to deal more actively with threats, but this impact is weaker in the presence of a strong traditional place attachment (de Dominicis et al., 2015). Given these findings, the current research defined the following hypothesis:

*H2*: Risk perception mediates the relationship between place attachment and coping strategies’ adoption.

*H2a*: Stronger active place attachment is associated with higher risk perception, which in turn is associated with more active coping strategies.

*H2b*: Stronger traditional place attachment is associated with lower risk perception so that, the lower the perceived risk, the more often passive coping strategies are adopted.

### 2.5. Place attachment, eco-anxiety, and coping strategies

The environmental literature shows that the role of emotions associated with climate change is increasingly being investigated because of not only these feelings’ relationship with well-being but also their effect on pro-environmental attitudes (Kurth and Pihkala, 2022). Individuals faced with prospective threats report emotions such as anxiety or anguish that affect the relationship between place attachment and intentions to deal with threats (Lemée et al., 2019). Potential dangers near people’s residences combined with their perceived inability to reduce threats effectively can trigger anxiety due to the anticipated negative events. These worries about climate change are defined in the extant literature as eco-anxiety arising from persistent concerns about climate change and related future uncertainties (Kurth and Pihkala, 2022).

Greater anxiety about climate change is, however, not necessarily related to the adoption of more sustainable lifestyles (Lemée et al., 2019). Several recent studies of climate change’s impact on mental health have suggested that eco-anxiety levels are progressively increasing (Hayes et al., 2018). High eco-anxiety is associated with reduced overall well-being (Ojala and Bengtsson, 2019), general suffering, fear, worry, guilt, hopelessness, and existential questions about future mortality rates due to climate change (Hickman, 2020; Panu, 2020; Pihkala, 2020). Emotions of grief and solastalgia may also be experienced when changes in the landscape occur due to climate change events (Moser, 2013; Phillips and Murphy, 2021). Scholars suggests that emotions can be influenced by past experiences, exposure to media or social norms, since individuals tend to engage with social referents and can also feel social pressure to act toward climate change (van der Linden, 2014; Ogunbode et al., 2022).

Not all negative emotions related to climate change have the same effects, and the literature on eco-anxiety is still unclear on how these feelings are connected to actions focused on reducing climate change. Negative emotions can trigger different responses fostering either disengagement from a perceived threat or active behaviors that lessen the threat (Stanley et al., 2021). On the one hand, higher eco-anxiety is associated with existential fears, feelings of insecurity, and defensive behaviors such as denial, which are often associated with depression. Kapeller and Jäger (2020) assert that, when exposed to quite dense information on climate change’s effects, people experience more anxiety, but, when associated with greater skepticism and a weaker environmental identity, they engage in denial behaviors.

On the other hand, eco-anxiety can manifest itself in “practical anxiety.” Individuals can be uncertain about how to respond to ecological threats and challenges and can ponder questions such as whether they should have more children due to future risks from climate change (Kurth and Pihkala, 2022). Practical eco-anxiety has been associated with a stronger motivation to engage in risk assessment and minimization (Kurth and Pihkala, 2022). Specific negative emotions linked to risk perception can foster attitudes that promote problem solving through active involvement with dangers. For example, people can seek more information about related subjects to understand and assess threats, which is connected with active coping strategies (Kurth and Pihkala, 2022). Various studies have shown that, if people discuss issues that they feel challenge their views and values (i.e., topics such as politics or civil society), those individuals who experience more anxiety become more involved and willing to learn about those controversial subjects (Valentino et al., 2008; MacKuen et al., 2010).

Still other research has underlined the importance of understanding eco-anxiety’s significant contribution to explaining social behaviors that reveal a desire to deal with climate change. That is, people who care about ecology and have low levels of anxiety get more involved in green policies and report being motivated to act in accordance with their beliefs (Verplanken and Roy, 2013). Moreover, people expressing deep distress tend to criticize the lack of good responses to climate change at all governance levels, but only the more informed and politically engaged contribute with concrete adaptative suggestions (Moser, 2013).

Varied social and cultural factors can strengthen or reduce the feelings triggered by negative stimuli. For instance, children are particularly vulnerable to adults and peers’ influence on their perceptions of and attitudes toward climate change (Ojala and Bengtsson, 2019). In coastal communities, place attachment also plays an important role in regulating person-environment relationships. Individuals actively engage in surveillance when faced with potential dangers to the place to which they are attached, becoming more aware of risks and exhibiting higher anxiety (Lemée et al., 2019; Clayton, 2020; Sullivan and Young, 2020). Climate change’s effects have been felt at a “slow” pace in the Aveiro region as they are not always easily observable, so the present study focused on eco-anxiety’s practical effect as a motivator or inhibitor of residents’ adoption of coping strategies rather than its impact on their mental health.

This research thus included the following hypothesis:

*H3*: Place attachment is associated with increased eco-anxiety, which in turn is linked with the adoption of coping strategies, so eco-anxiety has a mediator effect on place attachment and coping strategies’ relationship.

*H3a*: Stronger active place attachment is associated with greater eco-anxiety, which in turn is connected with more active coping strategies.

*H3b*: Stronger traditional place attachment is associated with lower eco-anxiety, which in turn is related to more passive coping strategies.

### 2.6. Place attachment, risk perception, eco-anxiety, and coping strategies

This investigation sought to understand how active and traditional place attachment is connected to active and passive coping strategies—through a sequential mediation by perceived risk and eco-anxiety. Previous research has looked at the link between place attachment, risk perception, and different types of coping strategies (Devine-Wright, 2011; Ruiz and Hernández, 2014; de Dominicis et al., 2015; Stancu et al., 2020; Sullivan and Young, 2020; Domingues et al., 2021), but few studies have examined the relationship between varied place attachment dimensions and coping strategy styles. Other investigations have also focused on the relationship between types of attachment and ways individuals cope with anxiety levels (Ruiz and Hernández, 2014; Stanley et al., 2021). However, to our best knowledge, only one research has explored the connection between people-place bonds, states of anxiety, risk perception, and coping strategies (Lemée et al., 2019). The cited study did not concentrate specifically on eco-anxiety, so a gap exists in the empirical research on how eco-anxiety helps or constrains the ways people deal with climate change risks (Kurth and Pihkala, 2022).

The extant literature reports that people with active place attachment are more involved with community initiatives and that these residents seek to be more informed and vigilant, which means they have a more realistic perception of their area’s imminent dangers. This risk perception can thus trigger negative emotions such as persistent concerns about environmental issues and future uncertainties (i.e., eco-anxiety), thereby contributing to attitudes that encourage solving problems (Kurth and Pihkala, 2022). People with traditional place attachment tend to resist and complain more about measures that imply changes, preferring to outsource responsibility and relegate threats to more distant times and places. By perceiving less risk, these individuals also enjoy lower eco-anxiety, which is associated with inhibition of action, and they are more likely to adopt more passive coping strategies in order to diminish the negative emotions triggered by, for instance, problem relativization.

Based on the theoretical findings discussed, the present study’s fifth hypothesis were formulated as follows:

*H4*: The relationship between place attachment and coping strategies occurs through a sequential mediation by risk perception and eco-anxiety.

*H4a*: Residents with stronger active place attachment report higher risk perception and eco-anxiety, and this relates to their use of active coping strategies.

*H4b*: Residents with stronger traditional place attachment report lower risk perception and eco-anxiety and this relates to their adoption of passive coping strategies.

### 2.7. Trust in responsible authorities

Most residents do not have specialized knowledge about climate threats’ uncertainties. The extant literature argues that, given any knowledge gaps regarding threats, individuals’ risk perception is based on how much they trust the organizations responsible for risk management (Terpstra, 2011). In addition, people tend to form their opinions based on the information given by trusted authorities (Cologna and Siegrist, 2020). Trust in authorities is the level of confidence inspired by administration agencies responsible for managing climate change risks (Siegrist et al., 2005).

Individuals who have greater trust in the responsible authorities feel less at risk than people who are less trusting (Cologna and Siegrist, 2020). Relying on the authorities is a way to cope with the cognitive complexity of threat assessment and decision making about mitigation or adaptation behaviors (Cologna and Siegrist, 2020). These authorities play an important role in large-scale risk management by introducing risk reduction and community adaptation policies and providing useful information and financial resources for protection measures’ implementation. Administration officials can thus potentially influence different behaviors’ adoption when people seek to deal with risk (Cologna and Siegrist, 2020).

The current research controlled for the effect of trust in authorities on coping strategies’ adoption. The final hypothesis was defined as follows:

*H5*: Trust in authorities is correlated with coping strategies.

*H5a*: Greater trust in authorities is linked with more passive coping strategies.

*H5b*: Less trust in authorities is associated with more active coping strategies.

**Figure 1:**
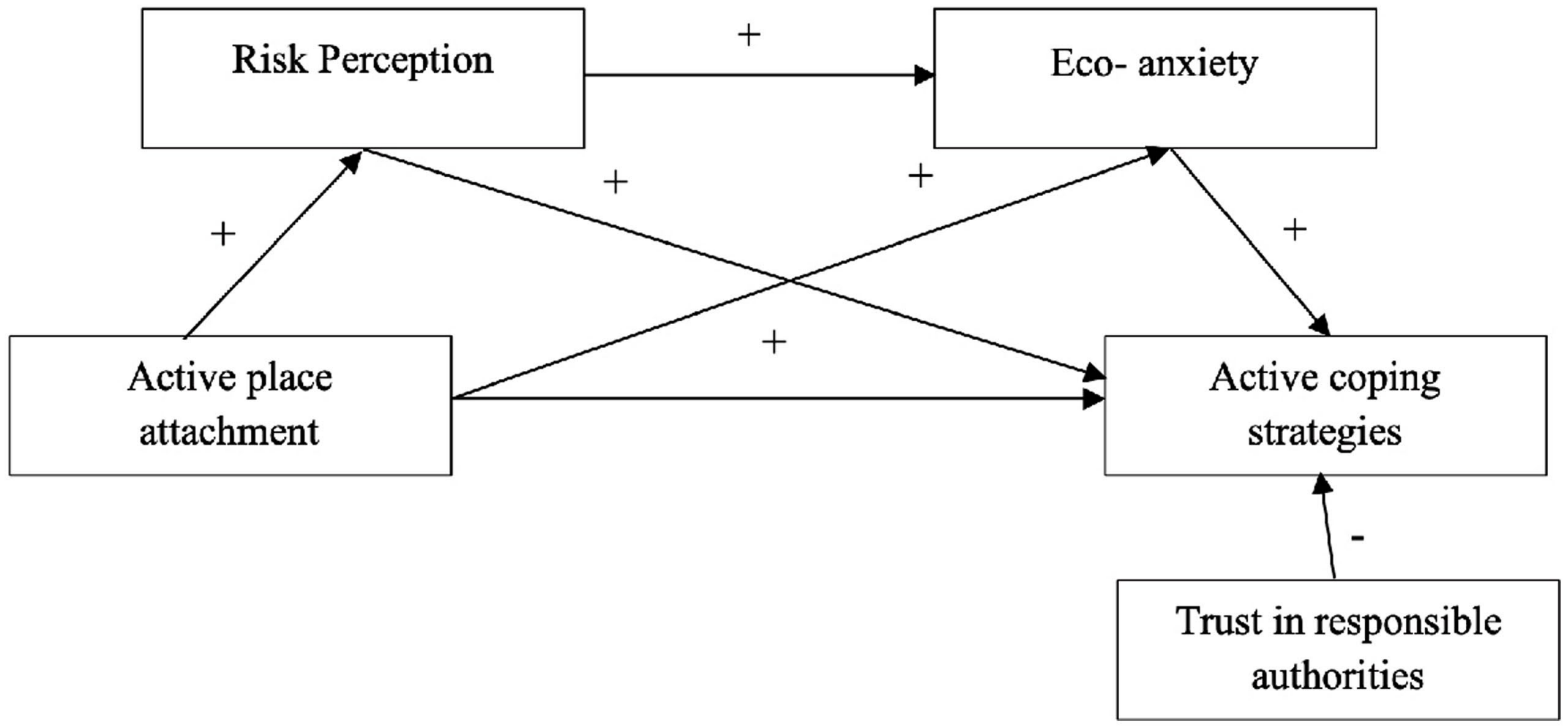
Sequential mediation model of active coping strategies.

**Figure 2:**
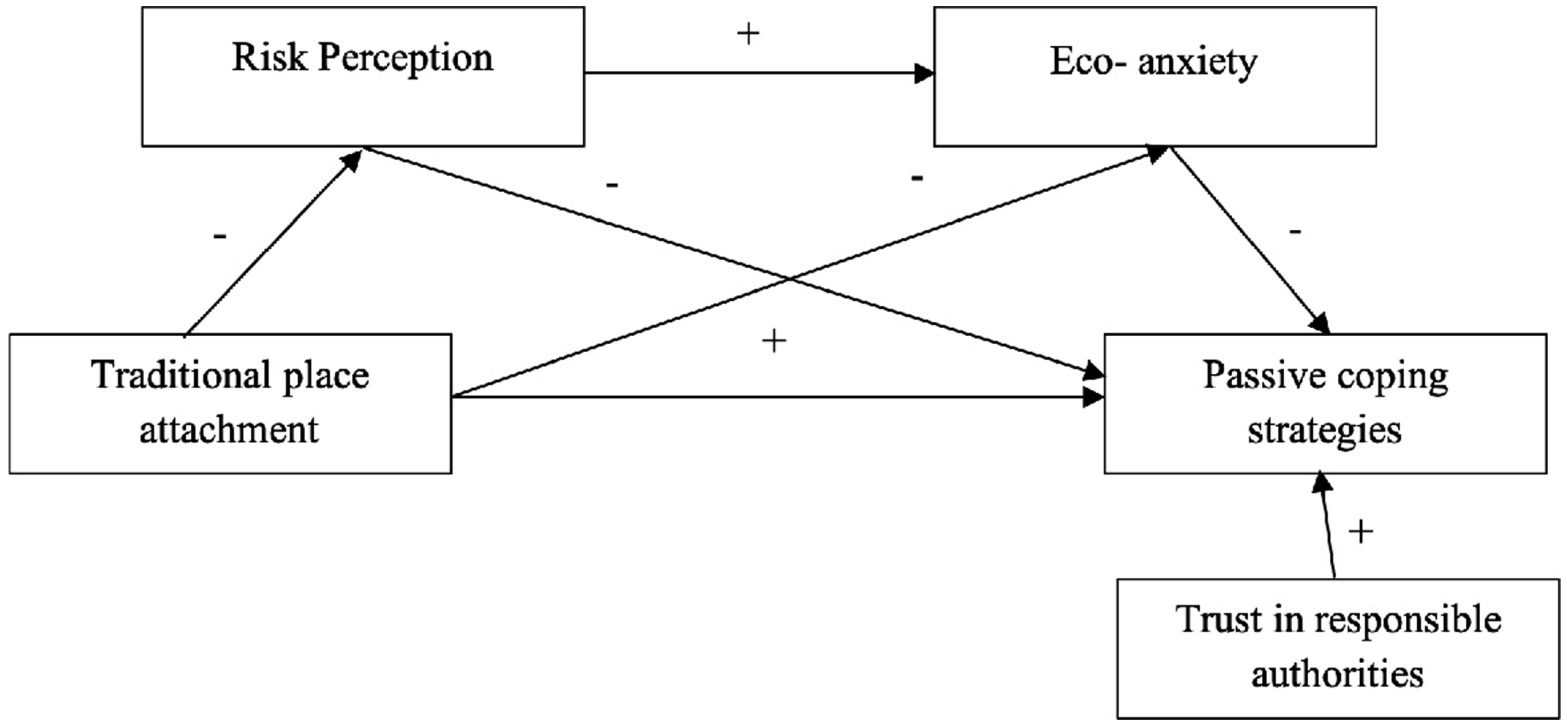
Sequential mediation model of passive coping strategies.

Figures 1, 2 present the two theoretical models proposed.

## 3. Materials and methods

### 3.1. Participants and procedures

The sample of this study consists of 197 participants recruited through informal processes (i.e., non-probabilistic convenience sampling). The inclusion criteria were that the respondents had to be at least 18 years old and live in the Aveiro district. Most participants were female (58%) with a higher education degree (71%). The mean (M) age was 39.35 years (SD = 9.96; min = 23; max = 68), and the average time residing in the Aveiro area was 32.11 years (SD = 14.03; min = 2; max = 66).

In addition, most respondents reported living close to one of the three areas that may be affected by rising water levels:

- Seacoast: 19% resided up to 5 km from the coast, 40% up to 10 km and 32.5% between 11 km and 20 km from the coast (*M* = 11.32; SD = 8.14; min = 0; max = 51).
- Ria de Aveiro: 49% resided up to 2 km from the Ria de Aveiro and 19% between 2 and 5 km away (*M* = 5.62; SD = 8.46; min = 0; max = 60).
- Vouga River/other: 21% lived less than 5 km from the Vouga River, and 50% indicated they resided between 5 and 10 km from the river (*M* = 16.71; SD = 16.17; min = 0; max = 80).

The data were collected with an online questionnaire created on the Qualtrics platform and distributed to Aveiro district residents via direct contact, email, or social media such as Facebook and Instagram. The participants were informed of the study’s objective and assured that their data would remain anonymous and confidential. After giving their informed consent, the respondents completed the questionnaire. At the end, these individuals were given more information about the research project and a link to the Aveiro municipal’s Plan for Adaptation to Climate Change. The study’s procedures were approved by the university’s Ethics Committee (Ref. 25/2022). Further details about the study’s procedures can be consulted at Parreira (2022).

### 3.2. Instruments

All measurement instruments were selected from the relevant literature, translated into Portuguese, and adapted to reflect the Aveiro region’s rising water levels. The scales’ items were randomly presented to the respondents to diminish any effects that order or tiredness might have.

#### 3.2.1. Types of place attachment (predictor)

Different types of place of residence attachment were assessed (i.e., the active and traditional dimensions) with a 16-item scale adapted from the City/Town/Village Attachment Scale (Lewicka, 2011). The responses were given on a Likert scale (1 = “Totally disagree”; 5 = “Totally agree”). Active place attachment was measured with six items (e.g., “I like to get involved in local affairs” or “I like to follow the changes that occur in my locality”) This dimension’s composite index has moderate reliability (Cronbach’s alpha [*α*] = 0.69). Traditional place attachment was evaluated with five items (e.g., “I cannot imagine leaving this locality forever” and “I have strong family connections to this locality”). Only four items were included in this dimension’s composite index, which has moderate reliability (*α* = 0.68).

#### 3.2.2. Risk perception (mediator)

The perceived risk of rising water levels in the surrounding area was assessed using the five-item risk perception scale developed by de Dominicis et al. (2015) and adapted for the present research to the context of rising water levels in Aveiro. The responses were given on a Likert scale (1 = “Not likely”; 5 = “Extremely likely”). The participants were asked to indicate their opinion about the probability of certain events occurring (e.g., “being affected by the rise in water levels” or “being harmed more easily than other people in the district”). The scale aimed to assess participants’ risk perception by evaluating their judgment about the likelihood of flood occurrence and consequences for the self and the place/others. This scale has high reliability (*α* = 0.86).

#### 3.2.3. Eco-anxiety (mediator)

Respondents were given 12 items of the Hogg Eco-Anxiety Scale – HEAS-13 (Hogg et al., 2021). These individuals indicated how often during the last 2 weeks they had felt bothered by the listed problems while thinking about climate change (e.g., global warming, rising sea levels, species extinction, or ocean pollution). Their answers were given on a Likert scale (1 = “Not at all”; 4 = “Almost every day”) with reference to the listed statements (e.g., “feeling anxious about your personal responsibility to help solve environmental problems” or “unable to stop thinking about future climate change and other environmental problems”). The scale’s reliability is high (*α* = 0.86).

#### 3.2.4. Coping strategies (criterion)

The participants responded to 17 items adapted for the present study from Homburg et al.’s (2007) Coping Measurement Models for Eight Coping Scales. The answers were recorded on a Likert scale (1 = “Not true”; 4 = “Totally true”). The original study validated a two-dimensional metastructure of active (i.e., focused on problem solving) versus passive strategies (i.e., focused on “deproblematization”). As recommended by Homburg et al. (2007), the subdimensions of resignation, positive thinking, and guilt denial were left out of the current research.

For active coping, the respondents evaluated two strategies. These were problem solving (e.g., “I try to obtain precise information about the rising water levels in my region”) and expression of emotions (e.g., “I feel angry when I see what is happening here due to the rise in water levels”). A composite index was created, which presents quite good internal consistency (*α* = 0.81).

For passive coping, the items referred to three strategies. These were relativization (e.g., “I think that in the near future there will be a solution to this problem”), self-protection (“When there is the possibility of rising waters, I reduce my activities outside the home”), and well-being (e.g., “When problems occur with rising water levels, I stay calm and stick to my normal routine”). An acceptable reliability score was obtained when only relativization and well-being were considered (*α* = 0.59).

#### 3.2.5. Trust in authorities (predictor)

The residents’ trust in the authorities responsible for managing environmental risks was assessed with six items based on Vaske et al.’s (2007) and Carlton and Jacobson’s (2013) research. The responses were given on a Likert scale (1 = “I do not trust them”; 5 = “I fully trust them”). The participants indicated their trust in local authorities regarding various actions (e.g., “effectively managing risks connected with rising water levels” and “preventing future situations”). This scale’s reliability is high (*α* = 0.96).

## 4. Results

The statistical data analysis was conducted using IBM’s SPSS Statistics 27 software. The first step was to generate descriptive statistics and correlations. The second was to test the sequential mediation models using PROCESS macro version 3.5 (Hayes et al., 2018).

### 4.1. Descriptive analysis and correlations between variables

Table 1 lists the means, standard deviations, and Spearman’s correlation coefficients for the selected variables. On average, the respondents reported low levels of both coping mechanisms, with more frequent higher implementations of passive strategies (*M* = 2.35; SD = 0.58) than of active strategies (*M* = 1.88; SD = 0.56). The participants also reported strong active place attachment (*M* = 4.05; SD = 0.45) and moderate traditional attachment (*M* = 3.44; SD = 0.79). The perceived risk of increased water levels was rated as moderate (*M* = 3.05; SD = 0.79), and there was a low report of eco-anxiety (*M* = 1.49; SD = 0.46). The respondents also expressed an overall low level of trust in the authorities responsible for dealing with rising water levels’ effects in the Aveiro district (*M* = 2.58; SD = 0.79).

**Table 1:** Correlation coefficients.

Variables	M	SD	1	2	3	4	5	6	7	8	9	10	11	12	13
1. Active coping	1.88	0.56	–												
2. Passive coping	2.35	0.58	−0.11	–											
3. Active place attachment	4.05	0.45	0.20^**^	0.02	–										
4. Traditional place attachment	3.44	0.79	−0.02	0.06	0.28^**^	–									
5. Risk perception	3.05	0.85	0.38^**^	−0.18^*^	0.19^**^	0.03	–								
6. Eco-anxiety	1.49	0.46	0.54^**^	−0.39^**^	0.09	0.01	0.33^**^	–							
7. Trust in authorities	2.58	0.97	−0.19^**^	0.17^*^	0.02	0.08	−0.20^**^	−0.15^*^	–						
8. Age	39.35	9.96	0.08	0.09	0.09	0.00	−0.11	−0.02	−0.02	–					
9. Residence tenure	32.11	14.02	0.10	0.05	0.08	0.29^**^	−0.07	0.03	−0.04	0.65^**^	–				
10. Gender	1.58	0.50	0.10	−0.14^*^	−0.02	0.02	0.20^**^	0.12	−0.02	−0.09	−0.07	–			
11. Education	3.68	0.53	0.05	0.00	0.09	−0.04	0.15^*^	−0.07	−0.17^*^	−0.18^**^	−0.26^**^	0.19^**^	–		
12. Distance to seacoast	11.32	8.14	0.00	−0.06	−0.07	−0.03	−0.29^**^	0.18^*^	−0.09	0.04	0.06	0.09	−0.04	–	
13. Distance to Ria de Aveiro	5.62	8.46	0.01	−0.25^**^	−0.09	−0.01	−0.20^**^	0.16^*^	−0.06	−0.14	−0.05	0.10	−0.16^*^	0.55^**^	–
14. Distance to Vouga River/other	16.71	16.17	0.02	−0.03	0.01	−0.03	0.15^*^	0.13	−0.14	−0.13	−0.09	0.18^**^	0.08	0.02	0.12

M, mean; SD, standard deviation; **Correlation significant at the 0.01 level (two-tailed); *Correlation significant at the 0.05 level (two-tailed); gender: 1 = male, 2 = female.

The correlations between the models’ variables reveal a non-significant relationship between active and passive coping. Active coping has a significant positive relationship with active place attachment (rho = 0.20; *p* < 0.01), risk perception (rho = 0.38; *p* < 0.01), and eco-anxiety (rho = 0.54; *p* < 0.01). Active place attachment is, in turn, positively associated with risk perception (rho = 0.19; *p* < 0.01), and risk perception is positively connected to eco-anxiety (rho = 0.33; *p* < 0.01).

No significant correlation was found between traditional place attachment and passive coping. Passive coping is negatively linked with risk perception (rho = −0.18; *p* < 0.05) and eco-anxiety (rho = −0.36; *p* < 0.01). The variables of gender (rho = −0.14; *p* < 0.05) and distance to Aveiro’s estuary (i.e., the Ria) (rho = −0.25; *p* < 0.01) were controlled for because they are significantly associated with passive coping mechanisms.

In addition, trust in authorities has a negative correlation with active coping (rho = −0.19; *p* < 0.01), risk perception (rho = −0.20; *p* < 0.01), and eco-anxiety (rho = *−0*.15; *p* < 0.05). The results also show that people who adopt more passive coping strategies also report higher levels of confidence in authorities (rho = 0.17; *p* < 0.05).

### 4.2. Sequential mediation analysis for active coping

The sequential mediation predicted by the theoretical models (see Figures 1, 2 above) was tested using Model 6 of PROCESS version 3.5 (Hayes et al., 2018). H1a proposes that active place attachment is associated with more active coping strategies, but, as Table 2 shows, this attachment dimension’s total effect is not statistically significant (beta coefficient [*B*] = 0.10; not statistically significant [n.s.]). Therefore, H1a was not supported.

**Table 2:** Testing of sequential mediation model for active coping.

	Risk perception		Eco-anxiety		Active coping	
	*B*	SE	*B*	SE	*B*	SE
Total effect						
Constant	–	–	–	–	1.64***	0.37
Active place attachment	–	–	–	–	0.10	0.09
Trust in authorities	–	–	–	–	−0.18*	0.41
					*F*(2,194) = 4.07; *p* < 0.01; *R^2^* = 0.04	
Direct effect						
Constant	2.09***	0.55	1.20***	0.30	0.49	0.33
Active place attachment	0.18**	0.13	0.01	0.07	0.03	0.08
Risk perception	–	–	0.26***	0.04	0.25***	0.04
Eco-anxiety	–	–	–	–	0.44***	0.08
Trust in authorities	–0. 18	0.06	−0.13	0.03	−0.05	0.04
	*F*(2,194) = 7.18; *p* < 0.001; *R^2^* = 0.07		*F*(3,193) = 6.94; *p* < 0.000; *R^2^* = 0.10		*F*(4,192) = 24.09; *p* < 0.000; *R^2^* = 0.33	

B, beta coefficient; SE, standard error; *p < 0.05; **p < 0.01; ***p < 0.001; *R*^2^ = coefficient of determination.

H2a stated that risk perception mediates the relationship between active place attachment and active coping mechanisms. The results confirm that active attachment significantly predicts risk perception (*B* = 0.18; *p* < 0.05), which in turn is significantly connected with active coping (*B* = 0.25; *p* < 0.001). The indirect effect is also statistically significant, which is further evidence of a mediation effect (*B* = 0.06; lower limit confidence interval [LLCI] = 0.01; upper limit confidence interval [ULCI] = 0.11) and which thus confirms H2a.

H3a posited that eco-anxiety mediates the relationship between active place attachment and active coping strategies. Eco-anxiety has a significant association with active coping strategies (*B* = 0.44; *p* < 0.001), but active place attachment has no significant impact on eco-anxiety (*B* = 0.01; n.s.). The indirect effect is, therefore, statistically non-significant (*B* = 0.00; LLCI = −0.07; ULCI = 0.09), and H3a was unsupported by the data.

H4a proposed that a sequential mediation exists between active place attachment, risk perception, eco-anxiety, and active coping strategies. The results indicate a statistically significant indirect effect is present (*B* = 0.03; LLCI = 0.00; ULCI = 0.06). This finding means that active place attachment is associated with more risk perception (*B* = 0.18; *p* < 0.05), which in turn increases eco-anxiety (*B* = 0.26; *p* < 0.001) and then strengthens the use of active coping strategies (*B* = 0.44; *p* < 0.001). These results provide empirical support for H4a (see Table 3).

**Table 3:** Indirect effects for sequential mediation model of active coping.

Indirect effect	Effect	BootLLCI	BootULCI
Total	0.09	−0.02	0.20
AA > RP > AC	0.06	0.01	0.11
AA > EA > AC	0.00	−0.07	0.09
AA > RP > EA > AC	0.03	0.00	0.06

BootLLCI, Bootstrap lower limit confidence interval; BootULCI, Bootstrap upper limit confidence interval; AA, active place attachment; RP, risk perception; AC, active coping; EA, eco-anxiety.

Finally, H5b stated that less trust in authorities is associated with more active coping strategies. The data analysis confirmed a direct connection exists between these variables (*B* = −0.18; *p* < 0.05), thereby confirming H5b. However, the link becomes non-significant when all the mediation model’s variables are considered (*B* = 0.05; n.s.). The model explains 33% of the variance in active coping mechanisms (coefficient of determination [*R^2^*] = 0.33; *F*[2.192] = 24.09; *p* < 0.000).

### 4.3. Sequential mediation analysis for passive coping

H1b posited that traditional place attachment is associated with more passive coping strategies. As Table 4 shows, the total effect is non-significant (*B* = 0.06, n.s.), thus H1b was not supported. In addition, H2b stated that risk perception mediates the relationship between traditional place attachment and passive coping strategies. The results indicate that no significant link exists between traditional attachment and risk perception (*B* = 0.00; n.s.), which in turn fails to predict more adoption of passive coping strategies (*B* = −0.08; n.s.). The indirect effect is also not statistically significant (*B* = 0.00; LLCI = −0.01; ULCI = 0.01), so H2b was not validated.

**Table 4:** Testing of sequential mediation model of passive coping.

Total effect	Risk perception		Eco-anxiety		Passive coping	
	*B*	SE	*B*	SE	*B*	SE
Constant	–	–	–	–	2.32***	0.24
Traditional place attachment	–	–	–	–	0.06	0.05
Trust in authorities	–	–	–	–	0.13	0.04
Gender					−0.13	0.08
Distance to Ria de Aveiro					−0.17*	0.00
					*F*(4,192) = 4.00; *p* < 0.01; *R^2^* = 0.08	
Direct effect						
Constant	3.02***	0.35	1.01***	0.22	3.00***	0.29
Traditional place attachment	0.00	0.07	0.04	0.04	0.07	0.05
Risk perception	–	–	0.27***	0.04	−0.08	0.05
Eco-anxiety	–	–	–	–	−0.28***	0.09
Trust in authorities	−0.20**	0.06	−0.12	0.03	−0.06	0.04
Gender	0.21**	0.12	0.04	0.06	−0.09	0.08
Distance to Ria de Aveiro	−0.16*	0.01	0.12	0.00	−0.16	0.00
	*F*(4,192) = 5.32; *p* < 0.000; *R^2^* = 0.10		*F*(5,191) = 5.04; *p* < 0.000; *R^2^* = 0.12		*F*(6,190) = 6.50; *p* < 0.000; *R*^2^ = 0.17	

B, beta coefficient; SE, standard error; *p < 0.05; **p < 0.01; ***p < 0.001; *R*^2^ = coefficient of determination.

H3b suggested that eco-anxiety mediates the relationship between traditional attachment and passive coping strategies. The findings include that traditional attachment has no significant impact on eco-anxiety (*B* = 0.04; n.s.), but higher eco-anxiety is associated with a fewer passive coping strategies (*B* = −0.28; *p* < 0.001). This indirect effect is, however, statistically non-significant (*B* = −0.01; LLCI = −0.05; ULCI = 0.02), so the mediation effect and thus H3b were not supported by the data.

H4b proposed a sequential mediation in which traditional attachment is linked to less risk perception, which in turn is correlated with low eco-anxiety and with more passive coping strategies. The results reveal a statistically non-significant indirect effect (*B* = 0.00; LLCI = −0.01; ULCI = 0.01), which means the sequential mediation was not confirmed, and H4b was not corroborated by the results.

The findings nonetheless suggest that a mediation relationship exists between risk perception, eco-anxiety, and passive coping strategies. In this case, risk perception is associated with increased eco-anxiety (*B* = 0.27; *p* < 0.001), which then has a negative impact on residents’ adoption of passive coping strategies (*B* = −0.28; *p* < 0.001).

**Figure 3:**
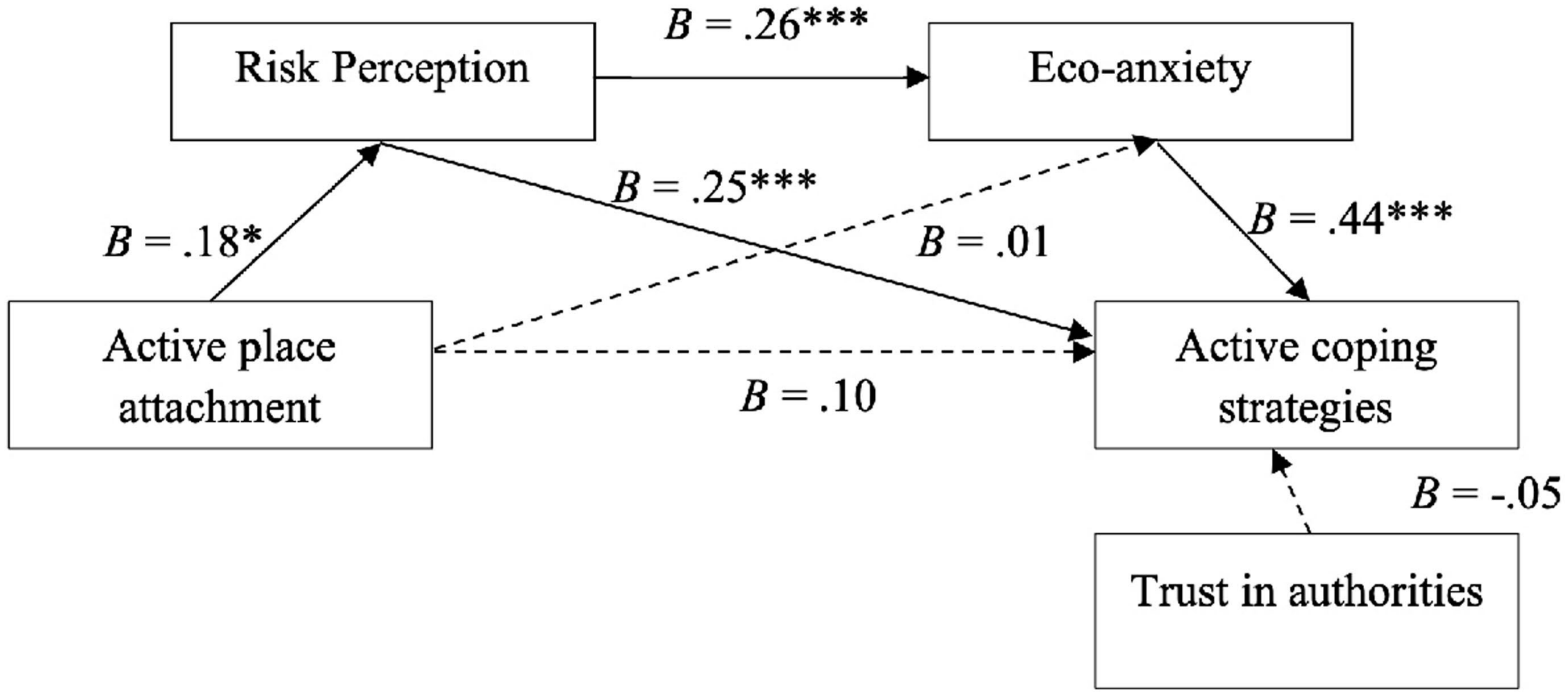
Sequential mediation model of active coping strategies through risk perception and eco-anxiety, controlling for trust in authorities (number = 197 participants).

**Figure 4:**
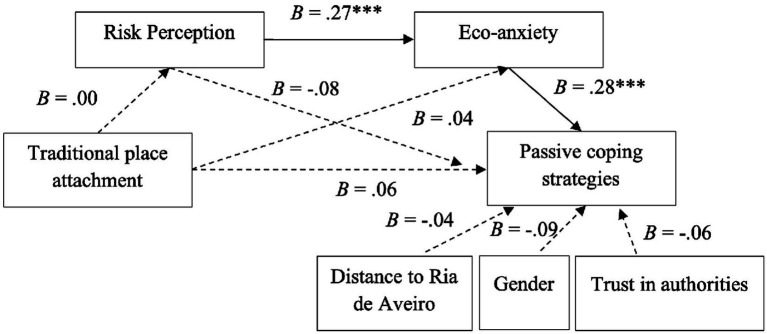
Sequential mediation model of active coping strategies through risk perception and eco-anxiety, controlling for trust in authorities, gender, and distance to Ria de Aveiro (number = 197 participants).

Finally, H5a posited that more trust in authorities is linked with more passive coping strategies. The results show that the effect is statistically non-significant (*B* = 0.05; n.s.), so H5a received insufficient support (see Table 5). The models explain 17% of the variance of passive coping (*F*[6,190] = 6.50; *p* < 0.000; *R^2^* = 0.17). Figures 3, 4 present the models’ main findings.

**Table 5:** Indirect effects of sequential mediation model for active coping.

Indirect effect	Effect	BootLLCI	BootULCI
Total	−0.01	−0.05	0.03
TA > RP > PC	0.00	−0.01	0.01
TA > EA > PC	−0.01	−0.05	0.02
TA > RP > EA > PC	0.00	−0.01	0.01

BootLLCI, Bootstrap lower limit confidence interval; BootULCI, Bootstrap upper limit confidence interval; TA, traditional place attachment; RP, risk perception; PC, passive coping; EA, eco-anxiety.

## 5. Discussion

Coastal areas are subject to the negative impacts of rising water levels that put communities and natural habitats at risk. Despite these threats, and because the dangers of higher sea levels often manifest themselves gradually, more communities are still located along the coast (Lloret et al., 2008). As residents vary in how they deal with and feel about water level threats, there is the need to better understand the cognitive and emotional factors that influence individuals’ choice of diverse coping responses.

The present study focused on communities dealing with rising water levels in the Aveiro district. The main goal was to analyze whether different types of place attachment are associated with contrasting coping strategies and whether these relationships are mediated by risk perception and eco-anxiety. The authorities have formulated strategies and plans for adaptation to or mitigation of climate change’s impacts on coastal regions, so the research model also considered the connection between residents’ trust in authorities and the coping strategies adopted.

The test of the proposed research models reveals that place attachment is not directly associated with either active or passive coping mechanisms. Place attachment’s role in residents’ choice of coping strategies appears to depend more on whether individuals perceive risk and feel some eco-anxiety, particularly in the case of active attachment. More active coping strategies thus imply that residents are aware of threats and their severity (Lazarus and Folkman, 1991; Navarro et al., 2021), and that they consider climate change to be a pressing issue. Active place attachment is associated with a greater awareness of local environmental threats, but, in the current research context, this recognition of risks may not be enough to motivate locals directly to adopt coping strategies.

Although Aveiro is considered an at-risk region, the current study was conducted outside any period involving imminent dangers or disasters, which may have contributed to weaker links between the model’s variables. Regardless, the results confirm that risk perception mediates the relationship between active place attachment and active coping strategies. As expected (Bonaiuto et al., 2016; Xu et al., 2018), active place attachment predicts greater risk perception, which in turn predicts more active coping mechanisms. The conclusion can be drawn that people who are more involved in the area where they live are more attentive to potential dangers and threats, and this awareness then leads to more active coping strategies.

In contrast, no evidence was found of risk perception’s mediation of the relationship between traditional attachment and passive coping mechanisms. Traditional place attachment seems not to determine the level of risk that people in Aveiro perceive regarding rising water levels, and the perceived risk is not related to adopting passive coping strategies. These findings differ from those reported by Stancu et al. (2020), yet these authors have focused on avoidant place attachment, which may be a less rooted attachment than the traditional type here examined. One possible explanation for the absence of the proposed relationship is that risk awareness seems to be related to higher involvement in local initiatives (Bonaiuto et al., 2016) and residents with higher traditional place attachment tend to be less socially active (Lewicka, 2013). Moreover, as perceived risk may hinder the type of attachment these residents have with their place (i.e., rootedness, not leaving) it is possible that they are less prone to consider such information as relevant in their daily life and, in turn, it would not trigger a specific coping behavior. Clarifying these results could also relate to how active and traditional attachment are linked. In this research, the two attachments are positively correlated, possibly because both types rate high on localism (Lewicka, 2013). Still, Lewicka (2013) showed that there can be cultural differences in how attachment types score on localism and activity, thus more cross-cultural research is needed on this subject.

The present investigation proposed that eco-anxiety mediates the link between place attachment and more coping strategies, but this empirical research did not validate the related hypotheses. A relationship was detected between eco-anxiety and more active coping strategies. However, place attachment has no direct impact on eco-anxiety for both active and passive coping mechanisms. This finding may indicate that eco-anxiety is triggered by residents’ surveillance of dangers to their area, so eco-anxiety is associated with the intensity of locals’ concerns (Lemée et al., 2019; Pihkala, 2020; Sullivan and Young, 2020) and this anxiety’s effect may be strongly dependent on risk perception.

In addition, the current results confirm a sequential mediation effect. People with stronger active attachment to their place of residence tend to perceive greater risk, which is related to greater eco-anxiety, and locals thus become more motivated to use active coping strategies to deal with perceived threats. Previous studies have similarly highlighted that individuals who actively engage in their neighborhood and community seek to be more informed about relevant risks and feel higher anxiety, which motivates them to adopt more active measures when dealing with these threats (Devine-Wright, 2011; Lemée et al., 2019; Sullivan and Young, 2020).

The present findings also reveal that risk perception can be enough to push people to implement active coping strategies, but eco-anxiety appears to be more closely associated with coping mechanisms than risk perception is. The sample’s data showed low eco-anxiety levels, a finding that could indicate that practical eco-anxiety is present (Kurth and Pihkala, 2022). Eco-anxiety may thus play a positive role that so far has been underresearched. Most studies of eco-anxiety have focused on its negative effects on mental health, yet the current results show that low eco-anxiety can motivate residents to use active coping strategies to deal with risks, such as trying to be more informed, participating in debates, or accepting and joining risk mitigation initiatives. Eco-anxiety can evidently be a moral emotion that suggests how much individuals care about important problems and uncertainties, which strengthens problem-solving attitudes (Kurth and Pihkala, 2022).

The current research further found that low eco-anxiety is positively related to both more active coping strategies and fewer passive coping mechanisms. These results indicate that eco-anxiety promotes more active ways of dealing with environmental problems and greater cognitive involvement in information collection, reorientation, and deliberation to make informed decisions. Eco-anxiety also increases responsiveness to uncertainties (Kurth and Pihkala, 2022) and prevents ruminating, deresponsibilization, and other passive strategies.

Contrary to expectations, the present study confirmed that the proposed sequential mediation is statistically non-significant for passive coping mechanisms. However, a mediating effect appears to exist for low risk perception, which leads to lower eco-anxiety and thus increases residents’ adoption of passive coping strategies. More research is needed to confirm this relationship.

Regarding trust in authorities, the results show that the less confident locals feel about the competence of the authorities responsible for solving problems, the more they tend to implement active coping strategies. This pattern may arise because residents who rely less on the authorities’ ability to manage and report the risks can perceive more risks, becoming more vigilant and actively engaging in resolving issues (Devine-Wright, 2011; Devine-Wright and Quinn, 2021). However, in the current final model, trust in authorities has no influence on other variables possibly due to risk perception and/or eco-anxiety’s mediation effect—a hypothesis that could be addressed in future studies.

### 5.1. Theoretical and practical implications

This empirical research sought to contribute to the existing knowledge by analyzing data on two place attachment dimensions’ impacts on different coping strategies. Much of the relevant literature focuses on place attachment as a one-dimensional construct, producing divergent results for its role in locals’ choice of ways to deal with environmental problems (Martins et al., 2009; Sullivan and Young, 2020; Devine-Wright and Quinn, 2021; Domingues et al., 2021). The present results indicate how residents’ different types of place attachment influence the way they cope with and feel about climate change threats, which emphasizes the importance of considering different attachment dimensions separately.

In other words, researchers must take into account that, for example, individuals who have lived in the same locality for a long time establish different types of place attachment. Scholars should also consider that active attachment can improve emotional regulation and trigger cognitive engagement processes that determine more surveillance of risks, which are significant when dealing with “slow-scale” threats such as rising water levels (Navarro et al., 2021). Different attachment dimensions should be considered as these influence how people deal with climate change, as well as having possible implications for cities’ future strategies and plans, especially regarding community-wide measures for adapting to climate change. Moser (2013) suggests that adaptation measures must be tailored to the social context and focus on engaging residents in their formulation and implementation. Actively involving people in search for adaptative responses can lead to greater engagement and may increase the resilience of coastal residents to climate change (Moser, 2013).

The current findings also add new knowledge about the driving role that eco-anxiety and low trust in authorities can play in individuals’ adoption of active coping mechanisms. The results indicate that the authorities responsible for implementing adaptation plans or communication and information strategies need to acknowledge that residents who demonstrate mistrust and eco-anxiety may more actively search for solutions. These residents are probably seeking to debate—and gain access to more credible information about—their area’s dangers and uncertainties (Terpstra, 2011; Kurth and Pihkala, 2022), and their involvement in discussions could result in an improvement and also greater acceptance of government measures.

Understanding the relevance of affective variables to how people deal with climate change can contribute to a fuller understanding of community behavior and to an improved design and implementation of local policies and coastal strategies. The latter may promote a better quality of life, particularly in regions where climate change’s impacts are more subtle.

### 5.2. Limitations and future implications

The present study had some limitations. First, the participants were recruited mostly via community groups’ social media networks, which tend to be used by people who actively engage in solving community problems. This sampling technique allowed more people to participate, but it may have produced a non-representative sample. For instance, the results showed that the inquired Aveiro community members have more active versus traditional place attachment. This tendency could be due to the participants’ recruitment through social networks, which may have favored people who like to get involved in community initiatives. Older longer-term residents with less education appear to establish a more traditional attachment to their area (*cf.* Lewicka, 2011), and they also tend to be less predisposed to filling out online or digital questionnaires. The data analysis further revealed that locals living closer to the Aveiro estuary and seacoast perceive more dangers that could affect them directly. This finding is not in line with the existing literature, which shows that coastal communities exposed daily to dangers feel less worried about risks in the long run (Martins et al., 2009; Luís et al., 2016; Domingues et al., 2021; Navarro et al., 2021). The present results could be explained by how residents’ proximity to the Ria de Aveiro seems to be associated with higher education levels. People with more education may try to be more informed about threats to their area, which contributes to stronger risk perception (Navarro et al., 2021). The sample size could also have been even larger to ensure the data analyses were more reliable and empirically robust.

Second, the place attachment and passive coping scales were found to have low reliability, so future research may want to use other more robust measures of these variables. The literature on eco-anxiety is quite recent and strongly focused on negative effects, which means that additional studies are needed to develop eco-anxiety scales that assess eco-anxiety’s less negative effects (Kurth and Pihkala, 2022). Further investigations could also examine eco-anxiety’s positive role in risk preparedness or climate change awareness campaigns to understand better how communication can trigger practical eco-anxiety in particular.

Notably, the measures applied were all self-reported, thereby restricting the data to how people perceive the ways they deal with threats and rather than their concrete actions. Given the specificities of coastal communities, the current results should be carefully analyzed before being generalized to other regions.

Future research may also need to examine the relationship of the proposed model’s variables specifically with passive coping mechanisms since no statistically significant results were obtained. The role of trust in authorities as a predictor or moderator variable can also be explored with regard to specific relationships. Studies of communication through digital channels can additionally analyze the effect that trust in the responsible authorities can have on individuals’ emotions or received information about climate change.

The models’ explained variance was low, so more research should be done to investigate other variables that may affect the relationships between place attachment styles and types of coping strategies, for example, place identity or coping mechanisms’ perceived efficacy (Lemée et al., 2019). Further research in the Aveiro district may also examine the relationship between place attachment and solastalgia (Phillips and Murphy, 2021), exploring its role in the acceptance of coastal adaptation policies. Longitudinal studies may also clarify changes in place attachment style (i.e., from active to traditional and vice versa), as well as how these shifts affect the coping strategies used by inhabitants of regions recovering from climate-related disasters.

## 6. Conclusion

Rapid behavioral change is urgently needed as the planet’s future and the human species’ destiny will be determined by people’s ability to adapt to climate change. Policymakers thus need to focus on understanding the factors and motivations that influence how individuals understand related threats and deal with risks, as communities’ cooperation is vital to formulating effective responses. This research highlighted the importance of analyzing multidimensional cognitive and emotional variables that can be associated with more active ways of dealing with climate change’s dangers. The results suggest that place attachment and eco-anxiety should be taken into account when developing communication strategies and change management plans seeking to ensure local communities’ active participation in finding solutions for adaptation to and mitigation of climate change’s effects.

## Data availability statement

The raw data supporting the conclusions of this article will be made available by the authors, without undue reservation.

## Ethics statement

The studies involving human participants were reviewed and approved by Iscte Instituto Universitário de Lisboa Ethics Committee. The patients/participants provided their written informed consent to participate in this study.

## Author contributions

NP and CM formulated the study’s plan and designed the data collection process. NP conducted the analyses and wrote the first version of the article. CM supervised the study’s development and revised the manuscript. All authors contributed to the article and approved the submitted version.

## Funding

This research was partially supported by Portugal’s Fundação para a Ciência e Tecnologia via grants UID/03125/2020, and contract DL 57/2016/CP1359/CT0004 awarded to the second author.

## Conflict of interest

The authors declare that the research was conducted in the absence of any commercial or financial relationships that could be construed as a potential conflict of interest.

## Publisher’s note

All claims expressed in this article are solely those of the authors and do not necessarily represent those of their affiliated organizations, or those of the publisher, the editors and the reviewers. Any product that may be evaluated in this article, or claim that may be made by its manufacturer, is not guaranteed or endorsed by the publisher.
